# Recurrent primary pyogenic ventriculitis in an adult woman: a case report

**DOI:** 10.1186/s12883-021-02422-2

**Published:** 2021-10-19

**Authors:** Shanyan Hong, Yingxia Yang, Qianying Zhang, Shitu Zhuo, Lingxing Wang

**Affiliations:** 1 Department of Clinical Nutrition, the Second Affiliated Hospital of Fujian Medical University, Quanzhou, 362000 Fujian China; 2 Department of Neurology, the Second Affiliated Hospital of Fujian Medical University, Quanzhou, 362000 Fujian China; 3 Department of Medical Imaging, the 910th hospital of the People’s Liberation Army, Quanzhou, 362000 Fujian China

**Keywords:** Recurrent, Primary, Pyogenic ventriculitis, *Escherichia coli*, Case report

## Abstract

**Background:**

Recurrent primary pyogenic ventriculitis has not been reported previously. We present a unique case of recurrent primary pyogenic ventriculitis in an adult. And we believe that our study makes a significant contribution to the literature.

**Case presentation:**

An adult woman with uncontrolled diabetes experienced two episodes of pyogenic ventriculitis caused by *Escherichia coli* over 4 years. She had typical imaging features, and the source of infection was undetermined. After antibiotic treatment, she recovered fully.

**Conclusions:**

Early recognition and therapy will improve patient prognosis.

## Background

Pyogenic ventriculitis is an infection of the brain characterized by suppurative fluid in the ventricles. It can have severe sequelae including death and often occurs secondary to external ventricular draining, abscesses, or intraventricular surgery. Reports of primary pyogenic ventriculitis are few, especially in adults. Moreover, there have been no reports of recurrent primary pyogenic ventriculitis in adults. Here, we present a case of recurrent primary pyogenic ventriculitis caused by the same bacteria in a Chinese woman.

## Case presentation

A woman experienced two episodes of primary pyogenic ventriculitis: the first was overlooked at another hospital, and the second was treated at our hospital. Despite having had diabetes for more than 10 years, the patient did not take oral antidiabetic drugs regularly or monitor her blood glucose level. She also had a history of right renal calculi combined with hydronephrosis, which she treated with traditional Chinese medicine and for which she underwent lithotripsy. She had no history of head trauma, chronic alcoholism, or cirrhosis.

### The first episode

**Fig. 1:**
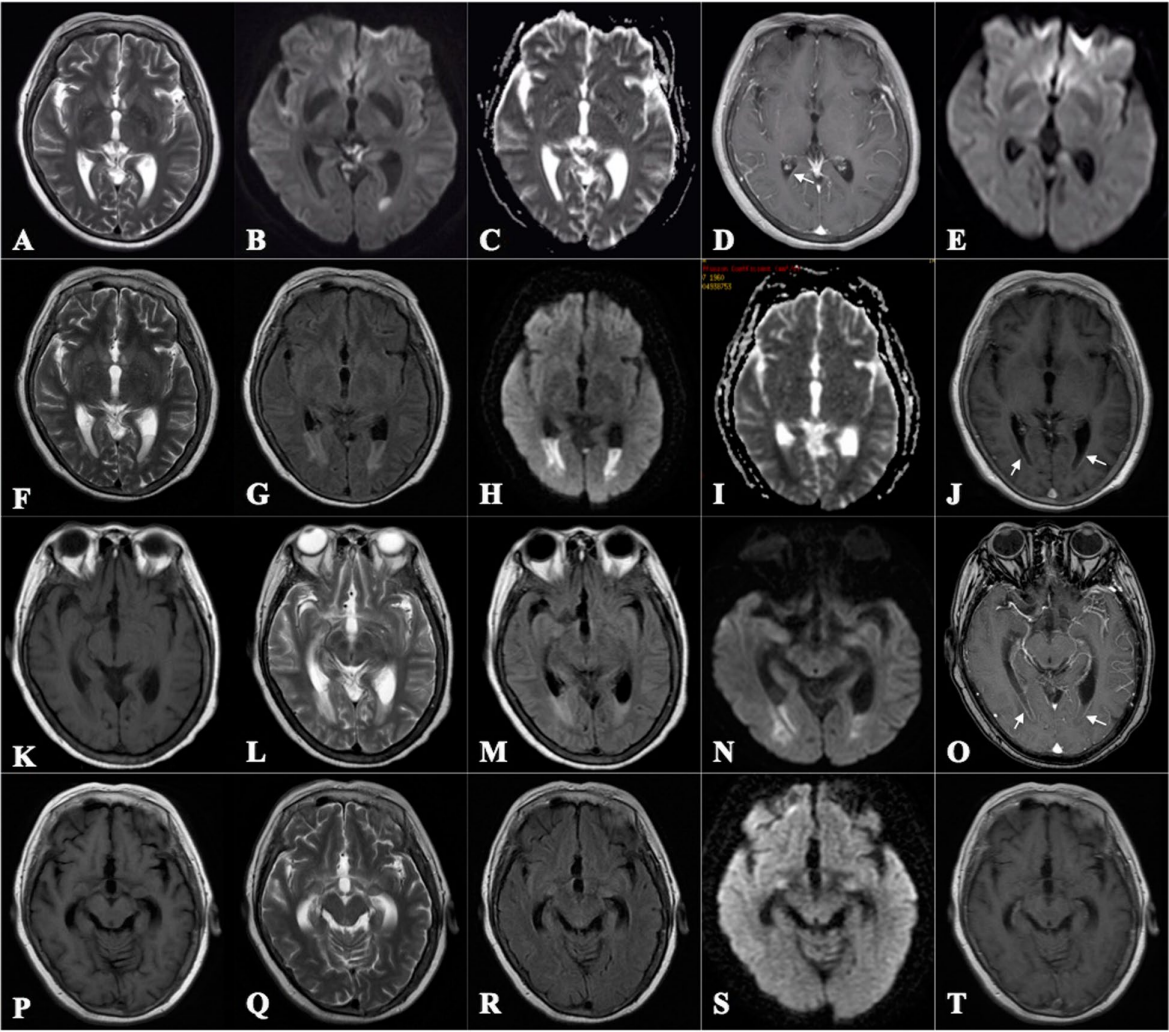
Brain magnetic resonance imaging performed at the previous hospital and our hospital. For the previous hospital, images at the time of admission (**A**–**D**) and 49 days after admission (**E**) are shown. For our hospital, images at the time of admission (**F**–**J**) and 25 (**K–O**), and 42 (**P–T**) days after admission are shown. Ventricular debris appeared as hypointense areas in the cerebrospinal fluid (CSF) on T2-weighted (**A**) and apparent diffusion coefficient (**ADC**) (**C**) images and as hyperintense areas in the CSF on the corresponding diffusion-weighted image (DWI) of the left occipital horn (**B**). Slight ependymal enhancement was found in the right occipital horn on the enhanced T1-weighted image (**D**). The hyperintensity in the left occipital horn was no longer apparent on DWI (**E**). T2-weighted (**F**) and ADC (**I**) images showed decreased intensity in the CSF in the occipital horn, whereas the corresponding fluid attenuation recovery (**G**) and DWI (**H**) images showed increased intensity with an enhanced ventricular line (arrow, **J**). The abnormal intensity in the bilateral occipital horns was reduced (**K–N**) but the ependymal enhancement was still obvious (arrow, **O**). The abnormal intensity had disappeared (**P**–**S**) and no enhancement was observed (**T**)

Four years ago, at the age of 56 years, the patient was diagnosed with bacterial meningitis at another hospital after experiencing fever and headache. At that time, she had an elevated white cell count (16.97 × 10^6^/L, 86.04% neutrophils) in a blood examination and pleocytosis (16,700 × 10^6^ cells /L, 88% multinuclear cells) with elevated protein (2.00 g/L) and decreased glucose (0.77 mmol/L) levels in a cerebrospinal fluid (CSF) analysis. Diffusion-weighted head magnetic resonance imaging (MRI) showed areas of hyperintensity in the CSF in the occipital horn of the left ventricle (Fig. 1B). T2-weighted (Fig. 1A) and apparent diffusion coefficient (Fig. 1C) images showed corresponding hyposensitivity in the CSF. *Escherichia coli* was detected in a CSF culture. After treatment with antibiotics [ceftriaxone at 2.0 g via intravenous drip (ivgtt)] once daily (qd) for 49 days, the cell count and protein level in the CSF decreased to 18 × 10^6^ /L and 0.51 g/L, respectively. Moreover, the hyperintensity on diffusion-weighted imaging (DWI) disappeared (Fig. 1E), and she recovered completely.

### The second episode

The patient, now 60 year-old, entered the emergency department of our hospital owing to abnormal behavior beginning 10 days earlier. During that time, she talked to herself and had a mild fever of 37–38 °C but no headache, vomiting, cough, or limb weakness. While in the emergency department, she had a generalized tonic-clonic seizure; 10 mg of diazepam was intravenously administered immediately, after which she did not have another seizure but became unconscious.

On admission to our hospital (hospital day 1), the patient had a blood pressure of 112/60 mmHg, a temperature of 37.5 °C, and a heart rate of 112 beats/min. When given painful stimuli, she was able to move both arms and legs but could not respond to oral commands or answer questions. Her pupils reacted to light. Neck stiffness was obvious. The deep tendon reflexes were weak, and the bilateral Babinski sign was positive. Her blood sugar and hemoglobin A1c levels were 13.95 mmol/L and 8.6%, respectively. Computed tomography (CT) of the head showed no lesions and chest CT excluded pneumonia. The white cell count was elevated (19.48 × 10^6^ /L, 89% neutrophils) in a blood examination. A human immunodeficiency virus test was negative.

Meningitis with diabetes but without encephalitis was considered. Ceftriaxone (2.0 g ivgtt qd), acyclovir [0.5 g ivgtt every 8 h (q8h)], and levetiracetam (0.5 g 3 times daily via feeding tube) were administered. On hospital day 2, a lumbar puncture was performed; the total cell count in the CSF was high (292 × 10^6^ /L, 59% multinuclear cells), with elevated protein (346.2 mg/dL) and decreased glucose levels. Blood and urine cultures were prepared. On hospital day 3, the patient still had a fever and high-throughput sequencing of the CSF showed the presence of *E. coli*. Fluid attenuation recovery (FLAIR; Fig. 1G) and diffusion-weighted (Fig. 1H) MRI indicated areas of high intensity in the CSF in the bilateral occipital horns of the ventricles. T2-weighted (Fig. 1F) and apparent diffusion coefficient (Fig. 1I) images showed corresponding areas of low intensity, with a partly enhanced ventricular lining (Fig. 1J).

The diagnosis was corrected to pyogenic ventriculitis. Ceftriaxone and acyclovir were discontinued and meropenem (2.0 g ivgtt q8h) was administered. A neurosurgical committee recommended continuous antibiotic treatment, with possible ventricular drainage if there was no improvement. The patient’s temperature began to drop. On hospital day 10, she became conscious and was able to answer questions but was slow in her reactions. The blood and urine cultures were negative. An abdominal ultrasound showed a right kidney stone of 5 mm in diameter with hydronephrosis. The results of an echocardiography examination were normal. The diagnosis was right renal calculi with hydronephrosis, and the urologist asked the patient to undergo kidney CT to determine the cause of the hydronephrosis. However, she refused, preferring Chinese medicine as her future treatment.

On hospital day 25, repeated brain MRI revealed relieved intraventricular empyema (Fig. 1K–O). Six weeks after admission, the patient was alert and exhibited no abnormal behavior or headache. In addition, the Mini-Mental State Examination (MMSE) score was 28/30 with 3 years of education. The abnormal signals on MRI had nearly disappeared (Fig. 1P–T), and a CSF analysis revealed that the cell count had decreased to 135 × 10^6^ /L and the protein level to 100.9 mg/dL. The antibiotic treatment was continued for 2 more weeks, after which the cell count in the CSF improved to 18 × 10^6^ /L. The patient was discharged and is in follow up. Additionally, the patient was advised to strengthen the control of diabetes by adhering strictly to antidiabetic medications and consult the urologist for further treatment of right renal calculi with hydronephrosis to avoid another episode. This case report did not involve animal or human studies and hence required no ethics committee approval.

## Discussion and conclusion

Pyogenic ventriculitis is similarly referred to as ventricular empyema, intraventricular abscess, ependymitis, or pyocephalus. We searched PubMed using these terms and excluded ventriculitis secondary to trauma, brain abscess, or neurosurgical procedures, such as intraventricular drain or ventricular catheter placement. We observed that most cases of primary pyogenic ventriculitis occur in children and neonates. Only 9 adult cases have been documented: 6 in a 2017 review [1] and 3 more since then [2–4]. There are no reports of recurrent primary pyogenic ventriculitis. To our knowledge, this is the first report of a recurrent case caused by *E. coli* in an adult.

Clinical presentations of pyogenic ventriculitis are non-specific and include headache, fever, seizure, focal neurological dysfunction, neck stiffness, and loss of consciousness. Patients are often misdiagnosed with meningitis or encephalitis, and the possibility of pyogenic ventriculitis is often ignored, as was twice done in our case (at the previous hospital and initially at our hospital). Meningeal irritation is often lacking in pyogenic ventriculitis. Neck stiffness was observed in only 1 of the 6 cases of primary pyogenic ventriculitis described by Gronthoud et al. [1]; other authors [3, 4] reported similar findings. In our case, the patient presented with obvious meningeal irritation, so antibiotics were administered immediately and a lumbar puncture was performed. However, in patients without meningeal irritation, antibiotic treatment may be delayed. Moreover, with pyogenic meningitis, the cell count in the CSF is usually higher than 1000 × 10^6^ /L, but on the patient’s second admission, the cell count was 292 × 10^6^ /L, which did not explain the severity of the MRI findings. We think that in cases of pyogenic ventriculitis, as the ventricles are the major location of infection, which has been proven in an animal model [5], the choroid plexuses are covered by debris, bacteria, leukocytes, and protein matrices, so the results of a CSF analysis may sometimes not accurately reveal the severity of infection in the ventricles. The changes in MRI should thus be taken into consideration.

MRI plays an important role in the diagnosis of pyogenic ventriculitis. The deposition of purulent fluid and debris in the ventricles leads to abnormal signals in MRI, including decreased signal intensity in T2-weighted and increased signal intensity in FLAIR/DWI images of the ventricles as opposed to the CSF; 94% of pyogenic ventriculitis cases showed ventricular debris in MRI [6], and this is regarded as the most characteristic feature of pyogenic ventriculitis. DWI is the most sensitive test for diagnosing pyogenic ventriculitis [7], and the MRI findings in our patient during her two episodes were clearly characteristic of pyogenic ventriculitis. Therefore, although she was only diagnosed with meningitis during her first admission, we could correctly diagnose her episode as pyogenic ventriculitis complicated with meningitis based on brain MRI. Moreover, during her second episode, an enhanced ventricular lining in the bilateral occipital horns was also found, indicating ependymitis; this was consistent with MRI changes in cases of ventriculitis and may be found in 60% of pyogenic ventriculitis cases [6]. Other specific MRI features of ventriculitis are hydrocephalus and hyperintense periventricular signals, which were not obvious in our patient. Since pyogenic ventriculitis is a severe intracranial infection, early recognition of characteristic changes in MRI will help to initiate proper treatment promptly and improve prognosis.

Primary pyogenic ventriculitis caused by *E. coli* is seldom reported [8–10], and no recurrent case has yet been presented. Ventriculitis caused by gram-negative bacterial infections often occurs after brain injury or surgery, when neurosurgical devices are used, and when immunodeficiency or organ dysfunction is present. However, none of these situations apply to our patient, which makes her recurrent infection puzzling. Her uncontrolled diabetes might be a predisposing factor, but how infection caused by the same bacterium could occur twice is unknown. Our patient had kidney stones combined with hydronephrosis, and upper urinary tract calculi are often associated with urinary tract infections [11]. Urinary tract infections have been reported as a distant cause of meningitis [8, 12], and *E. coli* is common in urinary systems. We thus speculate that the *E. coli* originated in the urinary tract. However, as the urine and blood cultures were prepared after antibiotic treatment, it is difficult for us to obtain further proof. Follow-up investigation should be done to reveal the source of the bacteria.

Besides ventricular drainage, antibiotics can be used to treat pyogenic ventriculitis. However, there is no definite guideline for antibiotic treatment of pyogenic ventriculitis. A duration of 6–12 weeks has been suggested [8], which is similar to the duration used for brain abscesses. During both of our patient’s infections, antibiotics were administered for 7–8 weeks. The prognosis of ventriculitis is dependent on the early initiation of antibiotic treatment and the reaction of the organism to the antibiotic. When the clinical response is not ideal, surgical intervention should be considered.

Our case is notable for its rarity and the same bacterial cause of both episodes. However, several lessons should be learned. The first episode was diagnosed as meningitis instead of ventriculitis, as was the second episode initially. This is concerning as it indicates the tendency to overlook the possibility of primary pyogenic ventriculitis and to perhaps underestimate the severity of the disease. Pyogenic ventriculitis may lead to hydrocephalus or even death, and effective treatment can reduce the possibility of the adverse result. Early initiation of long-term sensitive antibiotics use, surgery, or a combination of these is necessary once the diagnosis is established. Although the patient still had a good outcome during the first episode despite the missed diagnosis, we noticed that the antibiotic was used early and for a long duration coincidentally, according to the standard treatment guidelines for ventriculitis. Otherwise, the ventriculitis might be the potential source of infection even when meningitis was treated. And brain MRI is important and may aid diagnosis. Moreover, in primary ventriculitis, the ventricles are the major infection site, and changes in CSF may not always parallel the severity of infection. In cases of community-acquired bacterial meningitis, ceftriaxone is often the empirical antibiotic of choice; however, in severe pyogenic ventriculitis cases, ceftriaxone may not be effective enough, and exclusive dependence on CSF analysis results may lead to residual infections in the choroid plexus, which is a potential source of a recurrent episode. Lastly, in our case, blood and urine cultures were not prepared before antibiotic treatment began, which made it difficult to identify the underlying cause of the two episodes.

We present a case of recurrent primary pyogenic ventriculitis caused by *E. coli* with typical MRI features and a good recovery. Although the source of infection was unclear, uncontrolled diabetes may be a predisposing factor and the urinary tract may be the underlying source of the bacteria. Early recognition and therapy will improve patient prognosis.

## Data Availability

The datasets referenced for this study are available from the corresponding author on reasonable request.

## Abbreviations

CSF: Cerebrospinal fluid
CT: Computed tomography
DWI: Diffusion-weighted imaging
E*. coli.*: *Escherichia coli*
FLAIR: Fluid attenuated inversion recovery
Ivgtt: Intravenous drip
MRI: Magnetic resonance imaging
Qd: Daily
Q8h: Every 8 h
